# Phenocopy syndrome of frontotemporal dementia

**DOI:** 10.1192/j.eurpsy.2021.1123

**Published:** 2021-08-13

**Authors:** D. Martins, R. Faria, M. Pinho, S. Rodrigues

**Affiliations:** 1 Department Of Psychiatry, Hospital de Magalhães Lemos, Porto, Portugal; 2 Department Of Child And Adolescent Psychiatry, Centro Hospital e Universitário do Porto, Porto, Portugal

**Keywords:** frontotemporal dementia, Phenocopy syndrome, FTD phenocopy

## Abstract

**Introduction:**

Frontotemporal dementia (FTD) is a group of neurodegenerative disorders characterized by behavioral or language changes with progressive executive dysfunction. It´s subdivided into two variants, the behavioral and language variants. The phenocopy syndrome of frontotemporal dementia (phFTD) mimics the behavioral variant, but doesn´t show frontotemporal atrophy in neuroimaging and doesn´t progress to frank dementia over the years.

**Objectives:**

Presenting a review of phenocopy syndrome of frontotemporal dementia.

**Methods:**

Search on Pubmed® and Medscape® databases with the following keywords: “frontotemporal dementia and phenocopy” or “FTD phenocopy”. We focused on data from systematic reviews and meta-analyzes published in the last five years. The articles were selected by the authors according to their relevance.

**Results:**

Mutations in GRN and MAPT gens and genetic expansion of C9orf72 have been identified. The discovery of the C9orf72 expansion in psychiatric disorders (psychosis, bipolar disorder or depressive disorder), for some authors, represents that phFTD is a psychiatric pathology. In fact, there’s a higher frequency of psychiatric and psychological symptoms in phFTD compared to the variants of FTD. Usually are male patients who don´t have significant cognitive deficits, with preservation of executive functions and episodic memory. Until now, there are no definitive biomarkers of the disease. The prognosis is more benigne, unlike FTD, which has an average survival of 3 years since diagnosis.

**Conclusions:**

phFTD is a clinical and scientific challenge. The neurobiological bases remain unknown, requiring further studies in this field. The identification of markers that can differentiate patients with typical FTD and phFTD can facilitate prognosis orientation and pharmacological an non-pharmacological treatment.

